# Winners and losers: How the elevational range of breeding birds on Alps has varied over the past four decades due to climate and habitat changes

**DOI:** 10.1002/ece3.4838

**Published:** 2019-01-13

**Authors:** Luciano Bani, Massimiliano Luppi, Emanuel Rocchia, Olivia Dondina, Valerio Orioli

**Affiliations:** ^1^ Department of Earth and Environmental Sciences University of Milano‐Bicocca Milano Italy

**Keywords:** climate change, elevational distribution, habitat transformation, hierarchical partitioning, land abandonment, response curve

## Abstract

Climate warming and habitat transformation are widely recognized as worrying threatening factors. Understanding the individual contribution of these two factors to the change of species distribution could be very important in order to effectively counteract the species range contraction, especially in mountains, where alpine species are strongly limited in finding new areas to be colonized at higher elevations. We proposed a method to disentangle the effects of the two drivers of range change for breeding birds in Italian Alps, in the case of co‐occurring climate warming and shrub and forest encroachment. For each species, from 1982 to 2017, we related the estimated yearly elevational distribution of birds to the correspondent overall average of the daily minimum temperatures during the breeding season and the estimated amount of shrubs and forest cover. Using a hierarchical partitioning approach, we assessed the net contribution (i.e., without the shared effect) of each driver. Both temperature and shrub and forest cover showed a positive trend along the time series and resulted the most likely causes of the significant elevational displacement for 21 of the 29 investigated birds. While shrub and forest cover was found to be an important driver of the expansion of forest bird range toward higher elevations, the effect of temperature on favouring the colonization of previously climatically unsuitable forests at higher elevations was not negligible. Shrub and forest expansion resulted the main driver of the range contraction for edge and open habitat species, which suffered a distribution shrinkage at their lower elevational boundary. In light of climate warming, these results highlighted how the net range loss for edge and open habitat species, caused by shrub and forest encroachment consequent to land abandonment, should be counteracted by implementing proper conservation management strategies and promoting sustainable economic activities in rangeland areas.

## 1 INTRODUCTION

Although habitat transformation is still recognized as one of the major human drivers of species distribution changes at a global scale (Foley et al., 2005), a rising attention has been addressed in the last decades to the effects produced by climate change on biota (Parmesan & Yohe, 2003; Thuiller, 2007; Walther et al., 2002). In fact, the variation in climatic conditions forces species to adjust their spatial distribution according to their ecological niches, provided that resources are available (Thomas et al., 2004; Thuiller, Lavorel, & Araújo, 2005). Among the changes in climate features, those involving the raise of temperatures (referred as global warming) are certainly the best known and probably the most investigated. The magnitude of temperature change appears to be different in different geographical areas, and their effect can become more severe with increasing latitude or elevation (Loarie et al., 2009). Climate warming may affect species ecology with contrasting, additive or even synergic effects with the other co‐occurring processes (e.g., land‐use changes, pollution, harvesting, and species interactions; Mantyka‐Pringle, Martin, & Rhodes, 2013; Oliver & Morecroft, 2014). This is particularly noticeable in those areas where different relevant environmental changes act simultaneously. Species inhabiting medium latitude mountains, like the Alps, have been documented to suffer not only the effects of rising temperatures, but also those due to changes in agroforestry and pastoral practices, which have played an important role in shaping species’ distribution for a long time (Ausden, 2007; Maurer, Weyand, Fischer, & Stöcklin, 2006). Indeed, until a few decades ago, grazing by herds restrained the forest cover in mountain areas, but after the abandonment of pastures, shrubs and forests widely expanded, and even the tree line tended to raise in elevation, probably as a consequence of the synergic action of climate warming and land abandonment (Gehrig‐Fasel, Guisan, & Zimmermann, 2007; Leonelli, Pelfini, di Cella, & Garavaglia, 2011; Parolo & Rossi, 2008; Pernollet, Korner‐Nievergelt, & Jenni, 2015). In fact, the phenomenon was probably boosted by the increase in temperature, which limiting the period of snow cover, led to the colonization of the higher elevations by shrubs and forests (Gehrig‐Fasel et al., 2007). Overall, in the long‐term period, the abandonment of pastures and climate warming may promote the shrub and forest expansion at the expense of open habitat in mountain areas, leading to a general complex pattern that can vary between geographical areas (e.g., Rocchia, Luppi, Dondina, Orioli, & Bani, 2018).

As birds are particularly mobile and sensitive to environmental alteration, they could arguably be one of the first taxa to change its distribution in response to both climatic and habitat changes (Ambrosini et al., 2011; Both et al., 2004; Both & Visser, 2001; Visser, Both, & Lambrechts, 2004).

To quantify objectively the effects of gradual climate and habitat changes on species distribution, long time series of information are essential. Indeed, long time series may help to counteract the intrinsic, often wide, sources of variability and stochasticity, as well as the measurement and sampling noises contained both in biological and environmental data, which could make it difficult to find significant and strong relationships between them (Hilfinger & Paulsson, 2011). Of course, the data noise due to the stochastic errors of measurement of biological and environmental information can be difficult to manage without a long time series of data. On the other hand, the actual variability of biological data may be due to multiple environmental factors whose interactions are seldom taken into account (Guisan & Zimmermann, 2000).

The main purpose of this study was to investigate the drivers of the elevational range changes of breeding birds in Central Italian Alps, separating the effects produced by climatic (temperature increase) and habitat (shrub and forest recolonization) drivers. We did not take into account precipitations since they did not show a significant long‐term trend in the study area. We based our research on the 36‐year time series of breeding bird data in Lombardy (from 1982 to 2017), the longest data collection of breeding birds available in Italy, based on point count surveys (see the Methods section for further details). The variation in the elevational distribution of each species along the time was evaluated using a distribution model, which produced a yearly elevational distribution curve, in terms of presence probability, for a bird species along the elevational gradient. Then, we analyzed the contribution of climate and/or habitat changes in shaping the bird elevational range changes.

Finally, since species‐specific ecological traits are known to affect species responses to environmental changes (Copeland, Bradford, Duniway, & Butterfield, 2018; Dondina, Orioli, D'Occhio, Luppi, & Bani, 2017; Dondina, Orioli, Massimino, Pinoli, & Bani, 2015; Luppi, Dondina, Orioli, & Bani, 2018; Williams et al., 2010), we considered the species’ breeding habitat and migration habit as ecological traits potentially affecting the elevational responses of birds.

## 2 METHODS

### 2.1 Study area

**Figure 1:**
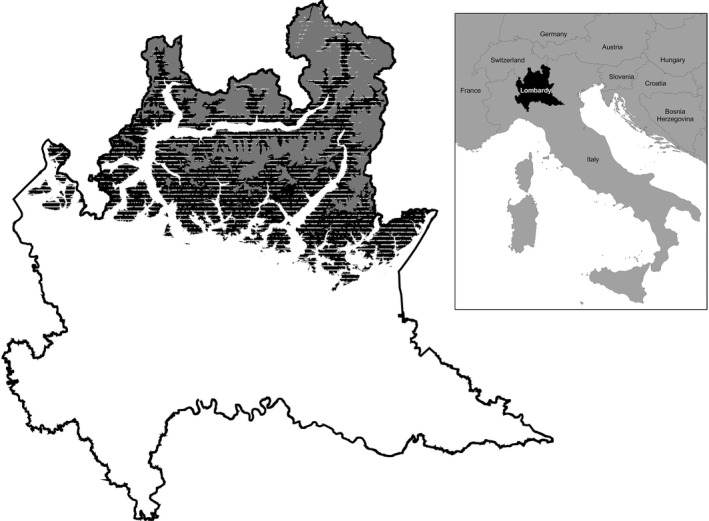
Study area (northern Lombardy). In black: area between 600 and 1,800 m asl; in gray: area above 1,800 m asl; dashed area: forest and shrub cover (source: DUSAF‐2015 cartography; ERSAF, 2010). The white area lays below 600 m asl or pertains to Apennines domain (southwestern corner) and was not investigated in the present study

The study was carried out in the Alps of Lombardy (Italy), above 600 m asl. The mountain area of Lombardy covers a large (160‐km wide) portion of the Italian Alpine chain, and includes the groups of the Lepontine, Rhaetian, and Orobic (or Bergamasque) Alps. The area surface amounts to 7,596 km^2^, about 45% (3,432 km^2^) of which is currently covered by forests, mainly located (92%) below 1,800 m asl (Figure 1). The highest peak (Punta Perrucchetti of the Bernina Massif) reaches 4,020 m asl and, overall, about 37% of the study area (2,822 km^2^) lays above 1,800 m asl. The climate is humid continental, characterized by a high seasonal temperature variation. In winter, the mean temperatures fall far below freezing at 2,000 m asl, while during summer, they can reach values of 10–12°C (RSY, 2015).

### 2.2 Sources of data

#### 2.2.1 Breeding bird data

Bird data for the period 1982–1988 were obtained from a dataset published by Realini (1988) concerning the mountain‐breeding birds of Lombardy, above 600 m asl. This is the first and only big dataset available containing historic data of breeding birds in the Alps of Lombardy. Bird data were collected in the field using the same point count technique adopted for the long‐term monitoring project of breeding birds in Lombardy started in 1992 (see below), from the last week of April to the first week of July. Since Realini bird data were published in geographical maps indicating each sampling point, we georeferenced all data. Data were found to be evenly distributed, both geographically and along the elevational gradient. Overall, they consist in 1927 point counts, but we did not considered data collected in 1988 (10 point counts only) in our analyses. Between 1982 and 1987, the mean number of point counts performed every year was 321.2 (range 67–782). Besides 1988, no data were available between 1989 and 1991, as the Realini research ended in 1988 and the long‐term monitoring project of breeding birds in Lombardy started in 1992.

Bird data for the period 1992–2017 were obtained from the dataset of the long‐term monitoring project of breeding birds in Lombardy (Bani, Massimino, Orioli, Bottoni, & Massa, 2009; Fornasari, Bani, De Carli, & Massa, 1998; Massimino, Orioli, Massa, & Bani, 2008). Data were collected using a standardized method based on the 10‐min, unlimited‐distance point count technique (Blondel, 1981; Fornasari et al., 1998). The technique is considered more effective in detecting bird species belonging to the orders Columbiformes, Cuculiformes, Apodiformes, Coraciiformes, Piciformes, and Passeriformes. Bird surveys were performed every year during the breeding season (10th May to 20th June), to minimize the count of migrants (birds not breeding in the study area) and to survey territorial birds. Censuses were conducted from sunrise to 11.00 a.m., only in good weather conditions, sunny to cloudy, without rain or strong wind (Bani et al., 2009). Overall, above 600 m asl (lower elevation threshold set by Realini), 5,140 point counts were performed, with a yearly mean of 233.6 (range 82–478), but no data were available for 1993, 1994, 1997, and 1998, when the long‐term monitoring project of breeding birds in Lombardy was interrupted due to the lack of funding.

The two projects provided a large amount of data collected over wide areas, but since they did not rely on multiple surveys in the same season, it was impossible to account for species detection probability. However, the large dataset used for this research should overcome the potential limit of imperfect detection, reducing the noise produced by stochasticity in species discovery (Dondina et al., 2017). In addition, Fuller and Langslow (1984) highlight that 10‐min point counts are satisfactory in detecting more than 70% of birds present at the census site and recommend how “counts exceeding 10 min are wasteful of field effort which could be used to improve other aspects of sampling”, such as the number of sampling units (see also Matsuoka et al., 2014).

In the long‐term monitoring project of breeding birds in Lombardy, data were collected as species abundances (number of individuals of each detected species per point count), while the Realini survey only recorded species presences in each point count. Thus, we merged the two datasets using information on species presences only.

Overall, the two projects provide 28 annual survey in the 36‐year time series starting from 1982, making a total of 7,067 point counts, ranging from 600 m asl to 2,700 m asl. The choice of the elevational bounds was due to sampling design constraints, the lower one due to historical data sampling design, the upper one because above this threshold the species’ presence drops abruptly in the study area. Overall, we surveyed 113 bird species breeding in mountain and alpine habitats, for a total of 58,080 bird occurrences.

#### 2.2.2 Habitat data

Habitat data came from to the digital cartography available for the study area in order to associate it with the time series of bird data. We wanted to evaluate the surface covered by shrubs and forests between 1982 and 2017 in the mountain area of Lombardy, above 600 m asl. For the analyses, we pooled together shrub and forest covers, since the distinction between the two land‐use classes using remote information is often difficult (Laliberte et al., 2004). However, the information obtained by merging the two classes together could be a good proxy of forest cover successional stages at the expense of open habitats. For land use, we use the DUSAF digital cartography (ERSAF, 2010) are available for the years 1980 (1:50,000), 1999, 2007, 2009, 2012, and 2015 (all 1:10,000; downloadable from http://www.geoportale.regione.lombardia.it/). To avoid possible bias in the evaluation of habitat data due to the different cartographies’ resolution, we resampled those with higher resolution to the lower resolution.

For the elevation, we used the Digital Terrain Model of Lombardy Region, with a 20‐m ground resolution (downloadable from http://www.geoportale.regione.lombardia.it/).

#### 2.2.3 Temperature data

Temperature data were obtained from the E‐OBS version 16.0 dataset with a resolution of 0.25 × 0.25 degree (see Haylock et al., 2008 for technical details), which can be downloaded for free at http://www.ecad.eu. Following the method proposed by Moreno and Hasenauer (2016), we downscaled the E‐OBS dataset of daily temperature to a 1‐km ground resolution, using the WorldClim 2 map (Fick & Hijmans, 2017). By doing this, we obtained a daily high‐resolution map for the annual series of temperature for the whole study area. For each of the 28 available years of breeding bird data, we built two annual temperature maps calculating the overall mean of the daily minimum temperature and the overall mean of the daily maximum temperatures recorded during the main local breeding season, respectively (May and June; see Bani et al., 2009). As the minimum and maximum temperatures were found to be strongly correlated (*R* = 0.907), all the analyses were performed considering only the overall mean of the daily minimum temperature of May and June.

The rationale of the selection of May and June temperatures is both (a) accounting for the effect of the yearly variability of temperatures during the breeding season on the yearly altitudinal distribution of birds (i.e., inter‐annual variability), and (b) accounting for the long‐term effect of temperature on the bird species altitudinal distribution (i.e., climate warming).

### 2.3 Annual elevational distribution curves of species

Since the data came from different projects, we were dealing with different sampling effort along the elevation gradient among years. This prevented a direct analysis of temporal changes occurred in the species elevational distribution, which would result biased among years according the year sampling efforts. Thus, we were forced to use a modeling approach to first assess the yearly species distribution, and subsequently, analyze its temporal trend. Indeed, for each species, we calculated the annual elevational distribution curve following the “curve response shape” method (Heegaard, 2002; Maggini et al., 2011), which defines a presence probability curve for a bird species along the elevational gradient. The curve was assessed as a smooth function (thin plate regression spline; Wood, 2017) of the elevation, other than northing and easting to account for spatial autocorrelation, using generalized additive models (Maggini et al., 2011; Wood, 2017). We set the maximum degrees of freedom to three for the elevation, in order to avoid overfitting and to obtain unimodal curves, and 15 for the space smooth (geographic coordinates; Maggini et al., 2011; Massimino et al., 2015). The analysis was performed using the *mgcv* package (Wood, 2003, 2011, 2017) in R version 3.4.3 (R Core Team, 2017).

Theoretically, up to five reference points characterize the curve: an optimum point (OPT; the central point of the curve), in which projection on the elevation axis indicates the elevation associated with the highest presence probability of the species; a central border left point and an outer border left point (CBL and OBL, two points at lower elevations), with a decreasing probability of the presence of the species; and a central border right point and an outer border right point (CBR and OBR, two points at higher elevations), with decreasing probability of the presence of the species. The central and the outer borders result from a fraction of the maximum response (Heegaard, 2002). The two central border points were defined by the projections on the elevation axis of the value densities corresponding to OPT*exp(−0.5), while the two outer border points by the projections on the elevation axis of the value densities corresponding to OPT*exp(−2). However, as we were working within a delimited elevational range, we could deal with truncated curves when some reference points fell outside the investigated range. Indeed, for several species, the distribution curve might fall under the 600 m asl or over 2,700 m asl.

For each year, bird occurrence data were bootstrapped (*n* = 200) and one curve for each bootstrapped sample was fitted in order to assess the estimated variability of the reference points. We considered a reference point estimate as reliable when the fitting procedure succeeded for at least 50% of the bootstrapped samples. Then, for each species and for each of its (up to 5) reliable reference points, we built a time series (reference points’ time series). Moreover, we considered the reference points having at least 10 reliable values along the time series as informative for the long‐term analysis.

### 2.4 Reconstruction of the habitat and climate dataset

Since an annual survey of the habitats is not expected within the study area, we built a habitat time series by interpolating the information contained in the available digital cartography. For each species, we calculated the yearly amount of shrub and forest cover within an elevational belt ranging from the minimum and the maximum value of elevation assumed by each of its reference points along the time series. Shrub and forest cover was interpolated using a thin plate regression spline, using a generalized additive model (Wood, 2017), and setting the maximum degrees of freedom to four for the year. Then, we made a yearly prediction of shrub and forest cover, thus building a habitat time series associated with the reference points’ time series (shrub and forest cover time series [km^2^]). Although annual habitat data were unavailable, we are confident that the information obtained from their interpolation should not deviate very much from the real condition, given the inertia of dynamics involving shrub and forest changes over large areas.

As regards climate data, for each species and for each of its reference points, within the elevational range defined by the minimum and the maximum value of elevation assumed by the same reference points along the time series, we calculated the overall average of daily minimum temperatures of May and June for each year (minimum temperature time series [°C]).

### 2.5 Elevational range of breeding birds and its trend over time

First, we assessed the presence of a temporal trend in the species’ reference points. To this aim, we fitted a weighted linear regression using the elevation value of each reference point as dependent variable, the year as independent variable, and the number of sampling units as weight. The analysis was performed using the *nlme* package (Pinheiro, Bates, DebRoy, & Sarkar, 2017) in R. Then, we classified the patterns of elevational range change for all species showing a significant trend in at least one of their reference points as: (a) “contraction” when the changes of the reference points brought to a distance reduction between the extreme reference points; (b) “expansion” when the changes of the reference points brought to an increase of the distance between extreme reference points; (c) “shift” when the changes of the reference points were consistent between each other. Depending on which reference points changed, we referred the observed change to the lower (OBL and CBL) or upper (CBR and OBR) reference points. If the slopes of the year pertaining to the two extreme reference points showed a significant trend with same sign but were significantly different between them, we evaluated if their change caused either a contraction or an expansion of species' range.

We have to acknowledge that reference points’ estimation using unlimited‐distance point count technique may be less accurate for open habitat species, since individuals observed at large distances (and different elevations) could be associated to the elevation of the point where observations were performed. Thus, due to the higher variance in observed elevation for open habitat species and the consequent estimation of their reference points, the long‐term changes in elevational distribution could result not significant (type II error). Moreover, the same kind of error could arise from the impossibility to take into account the imperfect detection that, however, should be quite limited according to Fuller and Langslow (1984).

### 2.6 Habitat and climate drivers of elevational range changes

In order to assess the environmental drivers of elevational distribution changes, we related the annual value of elevation of each reference point showing a significant trend (reference points’ time series) to the annual values of shrub and forest cover (shrub and forest cover time series, [SF]) and to the annual values of the corresponding temperature (minimum temperature time series, [tn]). For this purpose, we used a weighted multiple linear regression, using the annual number of sampling points as weight. In this analysis, we did not account for the possible bias that could be induced by temporal autocorrelation in the dependent variable because this relation may be due to the obvious temporal autocorrelation in the explanatory variables. The temporal autocorrelation was tested with the Durbin–Watson statistic (Durbin & Watson, 1950), performed using the *car* package (Fox & Weisberg, 2011) in R. However, in very few cases, we found a temporal autocorrelation in the residuals.

In order to assess the contribution of both environmental covariates (habitat and climate), we adopted a hierarchical partitioning approach (Chevan & Sutherland, 1991), using the *ecospat* package (Di Cola et al., 2017) in R. We assessed the net contribution of the habitat covariate (i.e., removing the shared habitat and climate contribution), the shared contribution of both environmental covariates, and the unexplained variation for the values assumed by each reference point along the time series (Randin, Jaccard, Vittoz, Yoccoz, & Guisan, 2009). Then, we classified the drivers as follow, based on their contributed deviance: (a) shared contribution >0.9, driver not distinguishable; (b) both net habitat and climate contribution ≥0.1: independent effect of both drivers; (c) net habitat contribution ≥0.1 and net climate contribution <0.1, habitat driver; (d) net climate contribution ≥0.1 and net habitat contribution <0.1, climate driver; (e) unexplained variation >0.9, neither habitat nor climatic driver.

For each species showing a significant elevational range change, we assessed the association of each pattern of elevational range change with the (a) breeding habitat, (b) the migration habit, and (c) the range change driver. The breeding habitat (open, edge, or forest species) and migration habit (long‐distance migrant [ldm], short‐distance migrant [sdm] or resident [res]) were assigned according to Sicurella, Orioli, Pinoli, Ambrosini, and Bani (2017) and integrated by the authors where necessary. The association between each pair of categorical variables was assessed performing a log‐linear analysis (Agresti, 1996) on the observed bird species’ frequencies from a contingency table obtained by crossing the pattern of elevational range change, the breeding habitat, the migration habit, and the drivers of range change.

## 3 RESULTS

### 3.1 Descriptive statistics of habitat and climate changes

**Figure 2:**
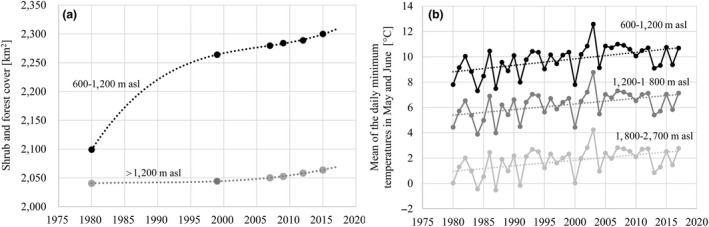
Descriptive statistics between 1980 and 2017 of (a) the shrub and forest cover [km^2^] and their trend (between 600 m and 1,200 and above 1,200 m asl), and (b) the average of daily minimum temperatures [°C] in the months of May and June and their trend in three elevational belts, in the Alpine areas of the Lombardy Region. For the shrub and forest cover, data were interpolated using a generalized additive model on available survey data (filled circles). For temperatures, data were derived from the E‐OBS dataset. See the text for details on data sources

In the Alps of Lombardy (above 600 m asl), the shrub and forest cover showed a positive trend (Figure 2a), switching from an overall cover of 4,140 km^2^ in the 1980 to 4,364 km^2^ in 2015 (+224 km^2^; +3.0% on the 7,596 km^2^ of the study area), with a more conspicuous increase between 600 m and 1,200 m asl respect to that observed above 1,200 m asl. Within the study area, also the temperature showed a clear positive trend over time (substantially invariant with respect to the elevation; Figure 2b), in accordance with the literature about climate warming in Europe (e.g., Beniston, 2006; Elguindi, Rauscher, & Giorgi, 2013). Indeed, the overall mean of the daily minimum temperatures in May and June increased from about 4.7°C in the eighties to about 6.0°C in the last 10 years, with an average increase of about 0.46°C per decade (see Klein Tank & Können, 2003).

### 3.2 Changes in the elevational range of breeding birds

Overall, we identified 125 reliable reference points for the analysis of the changes in the elevational distribution range for 29 mountain‐ and alpine‐breeding birds (Table 1). Forty‐nine reference points, pertaining to 21 species, showed a significant (*p* < 0.05) positive trend, which means that their elevational distribution significantly moved upward along the time series. The remaining 75 reference points did not show any significant variation along the time series (none showed a significant downward shift).

**Table 1:** Temporal trend of elevation of species’ reference points assessed by weighted linear regressions and corresponding pattern of range change for each species; *β*_y_ and *p*_y_: slope and level of significance for the year, respectively (left side of the table), and effect of forest and shrub cover and temperature as drivers of the elevation of species’ reference points (right side of the table)

Species	Breeding habitat	Migration habit	Reference point	Weighted linear regression^+^		Pattern of elevational range change	Weighted multiple linear regression					Contributed deviance				Range change driver
				*β*_y_	*p*_y_		*β*_tn_	*p*_tn_	*β*_SF_	*p*_SF_	*R*_adj_	Partial tn	Shared tn and SF	Partial SF	Unexplained	
Common Cuckoo *Cuculus canorus*	Forest	ldm	OBR	3.072	0.495	None										
			CBR	−1.819	0.818											
			OPT	−7.412	0.178											
Tree Pipit *Anthus trivialis*	Edge	ldm	OBR	1.807	0.668	Lower boundary contraction										
			CBR	2.867	0.266											
			OPT	5.052	0.001		18.47	0.134	46.42	0.001	0.520	0.042	0.414	0.307	0.237	SF
			CBL	8.842*	0.010		−4.417	0.767	96.46	<0.001	0.691	0	0.445	0.521	0.034	SF
			OBL	28.52*	<0.001		8.911	0.577	121.4	<0.001	0.800	0	0.866	0.130	0.003	SF
Water Pipit *Anthus spinoletta*	Open	sdm	OBR	−2.765	0.518	Lower boundary contraction										
			CBR	−5.796	0.374											
			OPT	−2.634	0.531											
			CBL	4.509	0.007		16.75	0.432	39.06	0.064	0.189	0.034	0.307	0.244	0.415	SF
			OBL	6.813	0.001		20.40	0.342	63.32	0.007	0.387	0.018	0.561	0.275	0.146	SF
Winter Wren *Troglodytes troglodytes*	Forest	sdm	OBR	9.904	0.003	Upper boundary expansion	−3.198	0.912	−112.1	<0.001	0.419	0	0.541	0.439	0.020	dde (SF)
			CBR	9.069	0.184											
			OPT	0.741	0.846											
			CBL	0.388	0.822											
Dunnock *Prunella modularis*	Edge	sdm	OBR	7.040	0.023	Lower boundary contraction	0.032	0.999	−75.98	0.006	0.327	0	0.401	0.485	0.114	dde (SF)
			CBR	7.682	<0.001		9.844	0.669	−78.77	0.002	0.455	0.002	0.597	0.328	0.073	dde (SF)
			OPT	6.944	<0.001		25.40	0.131	64.26	0.001	0.587	0.020	0.677	0.221	0.082	SF
			CBL	8.778	0.013		41.83	0.164	76.32	0.016	0.387	0.016	0.856	0.101	0.026	SF
			OBL	11.38*	0.004		43.68	0.116	103.8	0.001	0.565	0.002	0.946	0.049	0.003	nd
European Robin *Erithacus rubecula*	Forest	sdm	OBR	6.202	0.092	Upward shift										
			CBR	5.766	0.015		8.617	0.708	59.07	0.016	0.228	0.005	0.327	0.372	0.295	SF
			OPT	3.574	0.002		5.448	0.681	34.58	0.015	0.238	0.006	0.173	0.272	0.549	SF
			CBL	2.361	0.280											
Black Redstart *Phoenicurus ochruros*	Open	sdm	OBR	3.878	0.457	None										
			CBR	2.927	0.630											
			OPT	13.13	0.071											
			CBL	−0.115	0.982											
			OBL	0.391	0.944											
Northern Wheatear *Oenanthe oenanthe*	Open	ldm	OBR	−2.416	0.932	Lower boundary contraction										
			CBR	11.87	0.527											
			OPT	−4.767	0.247											
			CBL	9.626	0.186											
			OBL	11.01	<0.001		−19.04	0.463	127.0	<0.001	0.668	0	0.620	0.376	0.003	SF
Eurasian Blackbird *Turdusmerula*	Forest	sdm	OBR	10.41*	<0.001	Upper boundary expansion	73.84	0.021	68.81	0.042	0.314	0.102	0.808	0.061	0.029	tn
			CBR	12.69	<0.001		−0.276	0.989	126.8	<0.001	0.639	0	0.701	0.292	0.007	SF
			OPT	2.187	0.358											
Song Thrush *Turdus philomelos*	Forest	sdm	OBR	18.16	<0.001	Upper boundary expansion	33.45	0.506	−153.9	0.006	0.444	0	0.993	0.007	0	dde (nd)
			CBR	10.37	<0.001		20.93	0.530	80.20	0.026	0.314	0.005	0.762	0.188	0.045	SF
			OPT	3.692	0.004		16.07	0.390	30.73	0.127	0.164	0.049	0.259	0.175	0.517	SF
			CBL	−2.031	0.126											
Mistle Thrush *Turdus viscivorus*	Edge	sdm	OBR	1.047	0.641	None										
			CBR	2.176	0.184											
			OPT	10.12	0.124											
			CBL	8.715	0.175											
			OBL	9.862	0.183											
Lesser Whitethroat *Sylvia curruca*	Edge	sdm	OBR	3.758	0.393	Lower boundary contraction										
			CBR	17.71	0.140											
			OPT	5.313	<0.001		−4.179	0.833	59.30	0.004	0.563	0.001	0.441	0.391	0.167	SF
			CBL	36.64	0.054											
			OBL	15.29	0.046		58.65	0.118	60.56	0.063	0.495	0.020	0.927	0.035	0.018	nd
Eurasian Blackcap *Sylvia atricapilla*	Forest	sdm	OBR	9.373	0.041*	Upper boundary expansion	−51.69	0.328	−84.42	0.086	0.105	0.003	0.885	0.104	0.008	dde (SF)
			CBR	10.85	0.008*		12.14	0.680	130.7	0.000	0.496	0	0.820	0.176	0.004	SF
			OPT	1.376	0.446											
Western Bonelli's Warbler *Phylloscopus bonelli*	Forest	ldm	OBR	−1.019	0.737	None										
			CBR	7.309	0.249											
			OPT	1.000	0.730											
			CBL	−6.313	0.064											
Common Chiffchaff *Phylloscopus collybita*	Forest	sdm	OBR	11.06	0.050	Upper boundary expansion	26.53	0.382	−126.0	<0.001	0.524	0	0.916	0.082	0.002	dde (nd)
			CBR	7.639	0.009		75.18	0.012	13.86	0.649	0.177	0.722	0.099	0.008	0.170	tn
			OPT	4.570*	<0.001		49.70	0.001	24.88	0.093	0.482	0.253	0.484	0.048	0.215	tn
			CBL	4.487	0.027		68.71	0.004	1.281	0.952	0.315	0.462	0.325	0	0.212	tn
			OBL	−3.358	0.578											
Goldcrest *Regulus regulus*	Forest	sdm	OBR	5.174	0.166	None										
			CBR	3.346	0.070											
			OPT	1.240	0.155											
			CBL	0.476	0.797											
			OBL	3.772	0.246											
Common Firecrest *Regulus ignicapilla*	Forest	sdm	OBR	7.198	0.335	None										
			CBR	1.772	0.445											
			OPT	−1.098	0.194											
			CBL	−4.191	0.383											
			OBL	−2.418	0.137											
Spotted Flycatcher *Muscicapa striata*	Forest	ldm	OBR	6.678	0.359	None										
			CBR	22.26	0.303											
			OPT	0.623	0.860											
Willow Tit *Poecile montana*	Edge	res	OBR	11.37	0.015	Lower boundary contraction	0.771	0.987	−134.2	0.008	0.558	0	0.969	0.031	0	dde (nd)
			CBR	13.73	0.108											
			OPT	11.79	<0.001		−18.54	0.592	132.7	0.001	0.608	0	0.734	0.265	0.001	SF
			CBL	18.87	0.003		−28.41	0.775	196.4	0.065	0.182	0	0.861	0.139	0	SF
			OBL	17.06	<0.001		−74.76	0.169	221.3	0.001	0.611	0	0.728	0.272	0	SF
European Crested Tit *Lophophanes cristatus*	Forest	res	OBR	2.889	0.731	Upward shift										
			CBR	−3.481	0.613											
			OPT	5.693	0.086											
			CBL	11.18	0.010		34.71	0.140	170.2	0.018	0.404	0.109	0.182	0.526	0.183	tn + SF
			OBL	7.677	0.437											
Coal Tit *Periparus ater*	Forest	res	OBR	9.062	<0.001	Upper boundary expansion	102.4	0.005	−50.28	0.131	0.438	0.038	0.954	0.003	0.004	tn^a^
			CBR	6.947*	<0.001		66.13	0.001	47.46	0.009	0.608	0.094	0.823	0.038	0.045	tn
			OPT	3.698*	<0.001		43.57	0.001	18.93	0.119	0.508	0.285	0.407	0.041	0.267	tn
			CBL	2.632	0.043		27.04	0.130	18.50	0.295	0.159	0.126	0.251	0.056	0.567	tn
			OBL	4.015	0.127											
Eurasian Blue Tit *Cyanistes caeruleus*	Forest	res	OBR	10.94	0.022	Upper boundary expansion	20.73	0.539	106.0	0.014	0.388	0.002	0.861	0.114	0.022	SF
			CBR	24.24	0.414											
			OPT	2.481	0.130											
			CBL	1.722	0.405											
Great Tit *Parus major*	Forest	res	OBR	8.809	<0.001	Upper boundary expansion	48.13	0.035	68.61	0.006	0.499	0.038	0.824	0.089	0.048	SF
			CBR	7.256	0.035		59.64	0.089	44.40	0.221	0.238	0.099	0.783	0.040	0.078	nd
			OPT	−0.903	0.762											
Eurasian Jay *Garrulus glandarius*	Forest	res	OBR	10.57	0.059	Upward shift										
			CBR	10.69	0.164											
			OPT	6.491	0.017		12.61	0.632	51.60	0.092	0.232	0.012	0.502	0.222	0.263	SF
			CBL	8.480	0.119											
Hooded Crow *Corvus cornix*	Edge	res	OBR	1.485	0.762	None										
			CBR	1.093	0.819											
			OPT	0.980	0.757											
Common Chaffinch *Fringilla coelebs*	Forest	sdm	OBR	9.132	0.001	Upper boundary expansion	44.40	0.198	−62.19	0.070	0.244	0.039	0.784	0.103	0.074	dde (SF)
			CBR	7.384*	<0.001		23.57	0.130	65.66	<0.001	0.571	0.020	0.615	0.259	0.106	SF
			OPT	−0.840*	0.224											
Common Linnet *Carduelis cannabina*	Open	sdm	OBR	−1.221	0.832	Lower boundary contraction										
			CBR	0.172	0.972											
			OPT	3.082	0.211											
			CBL	6.007	<0.001		76.42	0.017	31.15	0.199	0.583	0.104	0.845	0.015	0.036	tn
			OBL	11.49	<0.001		19.28	0.629	119.3	0.003	0.672	0	0.978	0.022	0	nd
Common Redpoll *Acanthis flammea*	Edge	res	OBR	−1.231	0.648	Lower boundary contraction										
			CBR	2.448	0.299											
			OPT	5.257	0.005		27.06	0.310	45.78	0.043	0.506	0.026	0.688	0.152	0.134	SF
			CBL	10.42	0.002		23.47	0.511	95.47	0.005	0.652	0	0.945	0.051	0.003	nd
			OBL	13.21	<0.001		28.58	0.426	120.8	0.001	0.726	0	0.979	0.021	0	nd
Eurasian Bullfinch *Pyrrhula pyrrhula*	Forest	sdm	OBR	0.879	0.724	Upward shift										
			CBR	2.552	0.219											
			OPT	3.176	0.034		39.64	0.037	16.62	0.419	0.316	0.333	0.314	0.033	0.320	tn
			CBL	6.010	<0.001		25.49	0.064	48.44	0.003	0.681	0.045	0.674	0.188	0.093	SF
			OBL	3.659	0.278											

Notes

Effect of forest and shrub cover and temperature as drivers of the elevation of species’ reference points assessed by a weighted multiple linear regression, and relative estimation of covariates’ contributed deviance assessed by hierarchical partitioning approach; *β*_tn_ and *p*_tn_: slope and level of significance for the temperature, respectively; *β*_SF_ and *p*_SF_: slope and level of significance for the shrub and forest cover, respectively; *R*_adj_: adjusted *R*‐squared of the weighted multiple linear regression. Range change driver: tn: temperature; SF: shrub and forest cover; nd: driver not distinguishable; dde: density‐dependent effect (in brackets in the “range change driver” formerly deduced by the hierarchical partition approach).

a The *tn* was identified as the driver of the OBR reference point for the Coal Tit although should be set to *nd* following the criteria of the hierarchical partitioning approach (see the text for the explanation).

* Significant temporal autocorrelation at Durbin–Watson test.

Among the 21 species that changed their elevational distribution, eight showed an elevational range shrink due to a lower boundary upward contraction, four displayed an elevational upward shift, while nine showed an elevational range enlargement due to an upper boundary upward expansion.

We found a consistent upward displacement of the extreme reference points (OBL and OBR), for two edge species, the Dunnock (*Prunella modularis*) and the Willow Tit (*Poecile montana*), which should be classified as an “upward shift”. However, in both cases, we classified the elevational range change as a “lower boundary contraction” since the regression coefficients (*β*_y_; Table 1) of the OBL reference point was significantly higher than the regression coefficients of the OBR reference point, which indicates a net shrinkage of the elevational range for the two species. In fact, these two species seemed to partially compensate the loss of part of their lower range due to the disappearance of ecotones after forest and shrub encroachment by colonizing areas at higher elevations.

### 3.3 Shrub and forest cover and temperature as drivers of species elevational range changes

The contributed deviance of temperature in affecting the trend of reference point varied between zero and 0.722, while that of shrub and forest cover varied between zero and 0.526. The unexplained deviance was often small, higher than 0.3 in five cases only, and always lower than 0.567. The shared deviance (i.e., the common contributed deviance) of temperature and shrub and forest cover varied between 0.099 and 0.993, and was often high, being higher than 0.5 in most cases (35 out of 49; Table 1).

Among the 49 reference points that showed a significant positive trend along the time series, 29 were found to be affected by shrub and forest cover, 10 by the temperature, and one by both shrub and forest cover and temperature. Nine reference points were affected by a not distinguishable effect of shrub and forest cover and temperature (Table 1).

In five cases in which we found a statistical significance of the habitat driver, and in three cases in which the driver was not distinguishable, shrub and forest cover showed an opposite effect with respect to the known ecology of the species. Indeed, the upper reference points were significantly higher in elevation, the lower was the forest and shrub cover. Nevertheless, since the shrub and forest cover decreases with the elevation, the effect of the driver should be obviously spurious. Therefore, the upward displacement in elevation of the reference points could be due to other causes (temperature also showed a nonsignificant effect), which will be described in the Discussion section.

In the case of the Coal Tit (*Periparus ater*), the driver of the upward shift of the OBR reference point should be not distinguishable (nd) according to our hierarchical partitioning criteria. However, we identified “temperature” as a driver since the temperature effect was consistent with those of the lower reference points and the only significant covariate for the OBR reference point, while habitat cover showed an opposite effect on species ecology and was not significant in the weighted multiple linear regression (Table 1).

**Figure 3:**
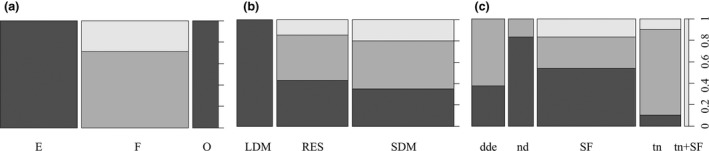
Proportion of bird species showing a pattern of range change (lower boundary contraction [Contr] in black, upper boundary expansion [Exp] in dark gray and upward shift [Shift], light gray), classified according to (a) habitat breeding preferences (edge [E], forest [F] and open [O]), (b) the migration habit (long‐distance migrants [LDM], residents [RES] and short‐distance migrants [SDM]), and (c) the range change driver (density‐dependent effect [dde], driver not distinguishable [nd], shrub and forest cover [SF], and temperature [tn]). The column width is proportional to the number of bird species belonging to the considered group

The log‐linear analysis highlighted a significant relationship between the pattern of elevational range change of birds and (a) bird's habitat; (b) bird's migration habit; and (c) range change driver. The statistical significance of these relationships is shown in Table 2, and the proportion of species for each association is indicated in Figure 3.

**Table 2:** Log‐linear models output of the observed species frequencies against all the combinations (main factors and interactions) of the categorical variables

	*df*	Deviance	Residual *df*	Residual deviance	*p*
Null			134	196.3	
Pattern of elevational range change	2	11.6	132	184.7	0.003
Bird's habitat	2	16.7	130	168.0	<0.001
Bird's migration habit	2	14.5	128	153.6	<0.001
Range change driver	4	29.7	124	123.9	<0.001
Pattern of range change: Breeding habitat	4	67.4	120	56.4	<0.001
Pattern of range change: Migration habit	4	11.1	116	45.3	0.025
Pattern of range change: Range change driver	8	19.7	108	25.6	0.011

## 4 DISCUSSION

The results of this research showed how the environmental transformations undergoing in mountain areas are leading to a shift toward higher elevations of a large part of the species living there (see also Rocchia et al., 2018; Ferrarini, Alatalo, & Gustin, 2017; but see Scridel et al., 2018). However, the upward shift was not only driven by temperature increase. Local processes, such as land‐use changes, also appeared to play an incontrovertible and likely synergistic role in exacerbating the displacement of species toward higher elevations. Although these evidences were largely derived from information on relatively common species, it is likely that even rare species pertaining to several different taxa, which are usually more demanding in terms of climatic and habitat factors, may be moving upward to a similar extent (e.g., Pernollet et al., 2015; Maggini et al., 2011).

Most of the bird species threatened by environmental changes in mountain areas were found to be open habitat species, which are mainly pushed upward by the loss of their physical space due to shrub and forest encroachment (Chamberlain, Negro, Caprio, & Rolando, 2013). This phenomenon is chiefly linked to the land abandonment caused by the widespread loss of economic performance of mountain pastoral activities (Schermer et al., 2016). Indeed, in a previous study conducted between 2006 and 2015, Rocchia, Luppi, Dondina, Orioli and Bani (2018) found that most of the breeding birds showed a significant change of their elevational range and that a large part of these birds were forest species. This pattern, as stated by the authors, is probably due to a significant upward shift of forests (i.e., forest expansion) during the period considered, since they did not find a significant positive trend for temperature.

In this research, which covered a long time period (1982–2017), we found a significant positive trend for temperatures in the Central Italian Alps, a trend that was virtually consistent at all elevation. At the same time, we also found a significant increase of shrub and forest cover, which was more conspicuous at lower elevations. Thus, the trends of the two covariates overlapped to some extent, making it difficult to disentangle the net effect of each driver on boosting the elevational changes in species distribution. However, the methodological approach adopted (i.e., hierarchical partitioning) allowed us to evaluate at least the partial (i.e., pure) contribution of each of the two drivers (i.e., the individual contribution without the joined contribution due to covariance). In addition, the availability of a long, almost continuous, time series of breeding bird data strengthened the power of statistical tests adopted in detecting the effect of the investigated drivers, even in the presence of possible stochastic noises, sources of uncertainty in bird data, and the possible effect of many other general or local factors affecting the elevational distribution of birds.

The physical habitat of an animal species is certainly a more important limiting factor compared with the local temperature, which can, however, be critical for the habitat to be colonized (Huey, 1991). For example, the local temperature may be considered a secondary limiting factor that could affect the colonization of a new area by a forest‐dwelling species, provided that there is an available forest habitat (primary limiting factor). Thus, it is not surprising that shrub and forest cover plays a primary role as a physical habitat in driving the elevational change of reference points for a large portion of the studied species. This point was widely confirmed by the upward shift of most of the lower reference points of bird species living in open or edge habitats, for which the encroachment of shrubs and forests (which is greater at lower elevations) led to a decrease of the available habitat, probably regardless of temperature increase. Moreover, for these species, we did not observe a corresponding upward shift of the upper reference points, which has consequently led to a net shrinkage of their elevational range, with a lower boundary contraction.

Conversely, forest species showed an upward shift with a displacement of one or more of their central reference points toward higher elevations. In other cases, they even showed an upper boundary expansion, with a displacement toward higher elevations of the upper reference points. These phenomena were likely due to the increase of shrub and forest cover occurred in the mountains within the region, and as highlighted several statistical significant relationships between the elevation values of reference points and this land use.

As expected, the habitat played the role of main driver. However, even the contribution of temperature was not negligible, especially for forest species, whose shift or expansion toward higher elevations was statistically significant. Temperature probably led to an improvement of climatic conditions in preexistent forest areas, which were previously not suitable. Indeed, the temperature driver was found to be more active in the upper part of the elevational range than the shrub and forest cover driver, which can be expected for species that usually inhabit lower and medium‐elevation forests, such as the Eurasian Blackbird (*Turdus merula*) or Eurasian Bullfinch (*Pyrrhula pyrrhula*). The lower reference points for these species were shifted upward by the habitat driver instead. For the European Crested Tit (*Lophophanes cristatus*), we found that the combination of temperature and shrub and forest cover drivers caused an upward range shift. Finally, the temperature was the only driver at all reference points for the Common Chiffchaff (*Phylloscopus collybita*) and the Coal Tit. In one case only, we found that temperature was a significant driver for an open species. This was noticeable since it was the only case in which temperature shifted the range of the Common Linnet (*Carduelis cannabina*) upward, causing a lower boundary contraction, without a pure statistically significant habitat effect.

Our research showed that the elevation increase of the upper reference points was in contrast with the ecology of forest species in several cases. In fact, it is important to note how, in those cases, the elevation of the upper reference points was inversely related to shrub and forest cover, without a positive temperature effect on the colonization of potentially suitable habitats at higher elevations. This pattern was recurrent, especially for the most common forest species, and seems to indicate that a specific process is in act. An upward shift in the reference points at lower elevations, induced either by an increase in habitat availability or by an improvement in the climatic condition due to temperature increase (which corresponds to a likely increase in population density), seemed to determine a sort of density‐dependent effect that may cause a colonization of even suboptimal habitats at higher elevations.

Species living in open habitats could also colonize preexisting suitable vacant areas at higher elevations, provided that these fall within their climate niche. However, many of these species had a truncated elevational distribution (i.e., lack of higher reference points), which suggested that they were already at the elevational limit reached by their habitats. Indeed, habitat formation is a slow process, especially at higher elevations, since the previous soil formation is likely to be slower than the speed at which the birds’ upward displacements could theoretically progress under environmental pressures (Edwards, Scalenghe, & Freppaz, 2007; Freppaz, Filippa, Caimi, Buffa, & Zanini, 2010; Körner, 2003).

Each of the observed patterns of range change was strictly associated with a particular bird ecological group and depended on a specific driver. Forest birds were found to colonize higher elevations shifting their range or expanding upwards, while birds living along edges or in open habitats suffered a contraction of their elevational range due to the shrinkage of the lower boundary. When effective, temperature was found to be the main driver affecting range expansion toward higher elevations for forest species. For many species suffering a range contraction, it was not possible to ascertain a pure contribution of one of the two investigated drivers, although a large portion of these species was found to be affected by shrub and forest encroachment. Finally, long‐distance migrants were all affected by a range contraction. Such a particularly unfavorable situation for these migratory birds can be emphasized by the “trophic mismatch”, as highlighted by different researches (Bairlein & Hüppop, 2004; Both et al., 2009; Pearce‐Higgins, Eglington, Martay, & Chamberlain, 2015).

In sum, forest species were clearly the winners in a context of shrub and forest encroachment in mountain areas, besides being favoured by an increase in temperatures. Conversely, edge and open habitat birds were the losers, due to the shrinkage of their habitat at lower elevations and the impossibility to find new habitats at higher elevations (but see Chamberlain et al, 2013). Considering the species that showed a lower boundary contraction (i.e., edge and open habitat species), the overall upward displacement of their reference points was about 390 m in 36 years. Conversely, the species that showed an expansion or an upward shift gained on average about 300 m in the same period of time. A similar rate of displacement was found for the Alpine Rock Ptarmigan (*Lagopus muta helvetica*) in Swiss Alps over a 29‐year period (Pernollet et al., 2015). However, these patterns are not universal and are probably more difficult to observe in shorter time periods (e.g., Archaux, 2004, Popy, Bordignon, & Prodon, 2010). Other taxa, usually represented by slow‐reacting organisms, such as tree species and bryophytes, also moved upward but with a less conspicuous displacement, about one order of magnitude lower (10–30 m per decade; Vittoz et al., 2013).

It should not be underestimated that the loss of open habitats in the long‐term, due to the abandonment of pastoral practices, could be further enhanced by climate warming. Indeed, a shorter permanence of the snow cover favors the progress of shrub and forest vegetation (Gehrig‐Fasel et al., 2007).

Both habitat transformation and climate warming are harsh threatening factors to biodiversity across the world, but while the first can be thwarted effectively from a local to a medium scale, the second requires shared and harmonized policies at a global scale (Goodstein, 2007).

In conclusion, how can we promptly and effectively counteract the negative trend showed by edge and open habitat birds in mountain areas? The conservation of open mountain habitats (grassland and prairies) is strictly linked to local ad hoc management practices that should also be implemented through several economic activities (e.g., marketing of local typical products, recreational outdoor activities, and food services) that depend on open habitats, even within the wide system of protected areas of the Central Italian Alps (Sicurella et al., 2017). Of course, these for‐profit activities should be addressed toward sustainable forms by adequate governance policies, in order to maintain a viable mountain economy in the long‐term, which should also be perceived as an attractive employment opportunity and produce a positive and broader perception in terms of ecosystem services (Schermer et al., 2016).

## CONFLICT OF INTEREST

None declared.

## AUTHOR CONTRIBUTION

L.B. and V.O contributed to the ideas and modeling framework. L.B. and V.O collected most of bird data. L.B., M.L, and E.R. conducted the modeling with insight from V.O. L.B, O.D., and V.O. wrote the paper with substantial contributions from E.R and M.L.

## Data Availability

N/A.
